# How Lebanese adults perceive travel vaccinations: A single center study

**DOI:** 10.1097/MD.0000000000050062

**Published:** 2026-07-31

**Authors:** Rania Sakr, Cima Hamieh, Mariana Helou, Rana Attieh, Alain Khouri, Remie Chrabieh, Ibrahim Ismail, Rola Husni

**Affiliations:** a Division of Family Medicine, Department of Internal Medicine, School of Medicine, Lebanese American University, Beirut, Lebanon; b Division of Emergency, Department of Internal Medicine, School of Medicine, Lebanese American University, Beirut, Lebanon; c Department of Internal Medicine, School of Medicine, Lebanese American University, Beirut, Lebanon; d Department of Dermatology, School of Medicine, Lebanese American University, Beirut, Lebanon; e Internal Medicine, Lebanese American University Medical Center Rizk Hospital, Beirut, Lebanon; f Division of Infectious Diseases, Department of Internal Medicine, School of Medicine, Lebanese American University, Beirut, Lebanon.

**Keywords:** travel, travel vaccine, vaccination

## Abstract

Travel vaccination is an essential public health intervention aimed at reducing the transmission of infectious diseases among international travelers. This study sought to evaluate the knowledge, attitudes, and practices of the Lebanese population about travel vaccination. This cross sectional single center study was conducted at the Lebanese American University Medical Center-Rizk Hospital. A structured questionnaire was administered to visitors and patients presenting to the outpatient clinics and emergency department between January 2020 and January 2021. A total of 1023 participants completed the questionnaire with 69.5% reporting that they have traveled within the previous year. Among these travelers, 41.1% usually carried a first aid kit, and 76.4% took emergency medications with them. Half of the participants considered pretravel medical consultation important, and 76.8% were aware of pretravel vaccinations. Concerns about vaccine side effects were relatively low, reported by 27.3% of participants. Concerns regarding vaccine adverse effects were relatively uncommon, with only 27.3% expressing such concerns. Regarding strategies to improve awareness of travel medicine, most participants (74.2%) identified social media as the preferred platform. Lebanese adults demonstrated relatively good awareness of travel vaccination and generally positive attitudes toward vaccination; however, pretravel consultation and preventive practices remained suboptimal. Improved travel health practices were associated more strongly with knowledge of travel vaccination and preventive medications than with attitude alone. These findings underscore a gap between awareness and implementation of recommended travel health measures.

## 1. Introduction

Nowadays, traveling has become easier, more affordable, and more widespread, enabling individuals to visit destinations across the globe. The World Health Organization (WHO) considers travel vaccination one of the most effective measures for preventing the spread of infectious diseases among travelers and across borders. The WHO further recommends that pretravel health assessments include destination-specific vaccine recommendations tailored to individual risk factors, underlying medical conditions, and the epidemiological profile of the destination. Accordingly, vaccinations help protect travelers against diseases that may be prevalent or endemic in the countries they visit, thereby contributing to improved global public health.^[1]^

The risk of acquiring hepatitis A or typhoid diseases was reported to be 60% and 70% lower, respectively, among vaccinated travelers than among unvaccinated travelers.^[2]^ Another study found that travel vaccinations can limit the spread of measles infection in the United States and highlighted the important role of vaccination campaigns in preventing the transmission of infectious diseases.^[3]^ Smith et al reported that only 4% of Australian travelers sought pretravel medical advice and vaccination before departure.^[4]^ Several factors may contribute to this low uptake, one of the most important being the variability of vaccination requirements across countries. Because each destination has its own disease-specific recommendations and entry requirements, travelers may find it difficult to determine which vaccines are needed before travel.^[2]^

Understanding public knowledge and perception regarding travel vaccination is essential for improving awareness and promoting preventive behavior. Although studies evaluating travel vaccination knowledge have been conducted in Europe, Australia, and North America, data from Middle Eastern populations remain very limited. Moreover, few studies have simultaneously evaluated validated measures of vaccine attitudes together with determinants of travel health practices. Therefore, this study aimed to assess the knowledge, attitudes, and practices of Lebanese adults regarding travel vaccination and identify factors independently associated with travel health practices. Although Lebanon is a small country, its emigration rate is increasing, with a 19% rise recorded between 2022 and 2023. At the same time, Lebanon receives a large number of tourists, particularly during the summer season.^[5]^ Therefore, Lebanon may represent a potential hub for the introduction and spread of infectious diseases, highlighting the importance of improving awareness of and uptake of travel vaccinations.

## 2. Materials and methods

### 2.1. Study design and participants

This cross sectional study was conducted at the Lebanese American University Medical Center–Rizk Hospital (LAUMCRH) between January 2020 and January 2021. The study was approved by the Lebanese American University Institutional Review Board (IRB No. LAUMCRH.RH2.7/Dec/2018). Adults presenting to outpatient clinics or the emergency department were invited to participate. Written informed consent was obtained from all participants. Participants were assisted in filling questionnaire.

### 2.2. Survey instrument

#### 2.2.1. Survey instrument

The questionnaire comprised 2 sections. The first collected demographic data, including age, education, occupation, healthcare-worker status, area of residence, and family status. The second assessed knowledge of and attitudes toward vaccination, with a focus on travel vaccination. Items were adapted from previously published questionnaires evaluating vaccination knowledge in different populations. The full questionnaire is provided in Appendix 1, Supplemental Digital Content 1.

Content and face validity were assessed by physicians with expertise in family medicine, infectious diseases, and travel medicine. The instrument was then piloted in a small sample representative of the target population to assess clarity, comprehension, and completion time. Minor wording revisions were made before study implementation.

#### 2.2.2. Sample size

Assuming a 3% margin of error, 95% confidence level, and a target population of 5 million, the minimum required sample size was estimated at 998 using Epi Info. A total of 1023 participants completed the questionnaire.

### 2.3. Statistical analysis

Data was analyzed using descriptive statistics, scale validation, bivariate screening, and multivariable regression. The results of the survey questionnaires are presented as raw counts and crude frequencies. Age groups were divided into 3 categories: <30, 30–50, and >50 years old. Relationships between the demographic categorical variables and knowledge, attitude, and practice were analyzed using the chi-squared test; a *P*-value < .05 indicated a significant difference. All statistical analyses were conducted in R (version 4.0.2; R foundation for statistical computing). Regression assumptions were evaluated before model fitting. Multicollinearity was assessed using variance inflation factors, while model calibration and goodness-of-fit measures were examined where appropriate. Adjusted odds ratios (aORs) with 95% confidence intervals were reported for logistic regression analyses. The excel database is in Appendix 2, Supplemental Digital Content 2.

## 3. Results

### 3.1. Participant characteristics

**Figure 1.**
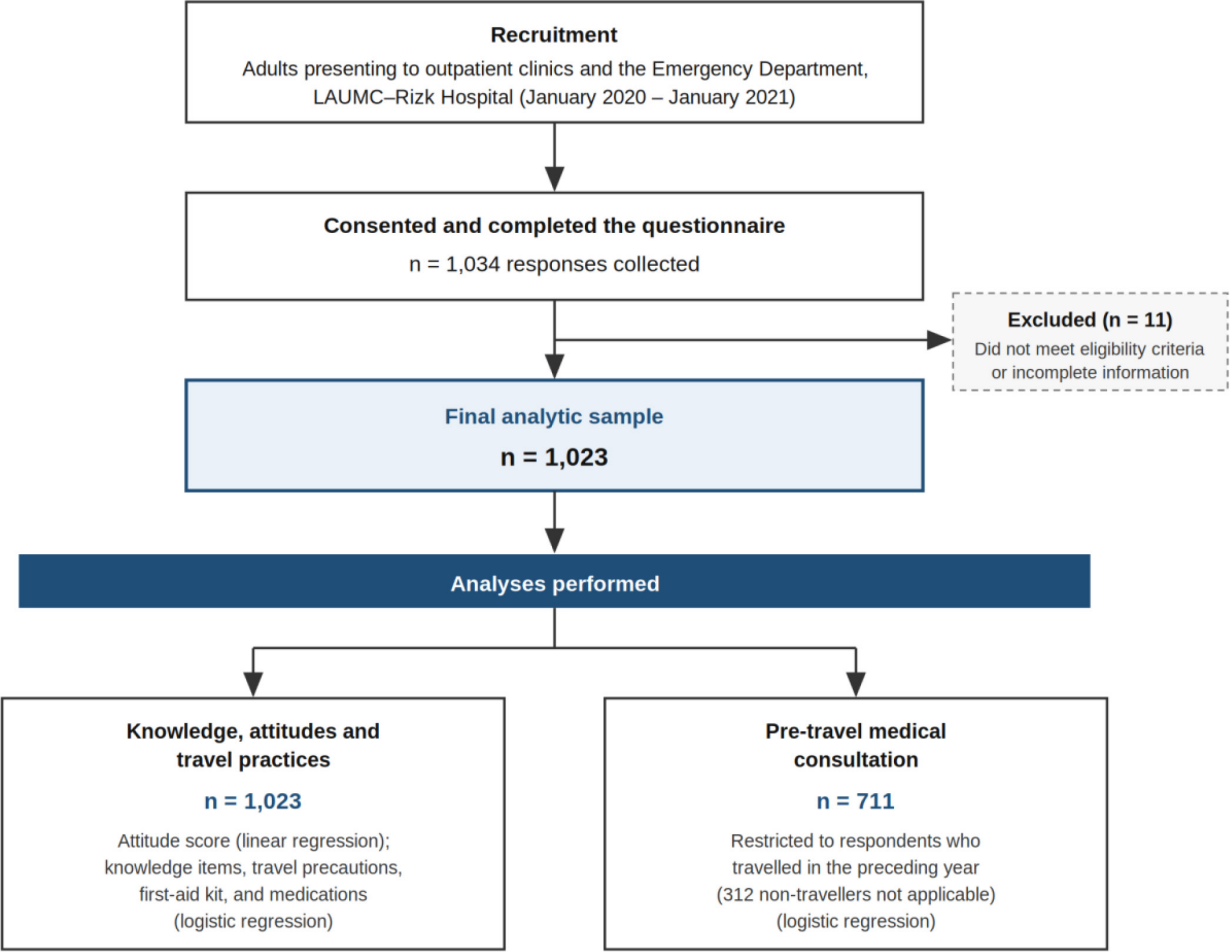
Participant flow diagram. LAUMCRH = Lebanese American University Medical Center.

The demographic characteristics of the 1023 respondents are summarized in Table 1 and illustrated in Figure 1.The sample was young (mean age 31.4 years, SD 11.5; 66.6% aged 18–30), predominantly female (56.5%), highly educated (70.7% graduate or postgraduate), and overwhelmingly Lebanese (96.3%). Healthcare workers comprised 27.6% of respondents.

**Table 1:** Sociodemographic characteristics of the study sample (N = 1023).

Characteristic	n (%)
Age, yr – mean (SD)	31.4 (11.5)
Age, yr – median (IQR)	27 (24–34)
18–30	681 (66.6)
31–45	208 (20.3)
>45	134 (13.1)
Gender – male	445 (43.5)
Gender – female	578 (56.5)
Education – school	72 (7.0)
Education – undergraduate	227 (22.2)
Education – graduate	470 (45.9)
Education – postgraduate	254 (24.8)
Residency – Beirut	436 (42.6)
Residency – Mount Lebanon	385 (37.6)
Residency – Bekaa	109 (10.7)
Residency – North	49 (4.8)
Residency – South	44 (4.3)
Occupation – healthcare worker	282 (27.6)
Occupation – general population	741 (72.4)
Marital status – single	674 (65.9)
Marital status – married	328 (32.1)
Marital status – divorced	13 (1.3)
Marital status – widowed	8 (0.8)
Has children – yes	267 (26.1)
Nationality – Lebanese	985 (96.3)
Nationality – other	38 (3.7)
Medical risk factors – none	879 (85.9)
Medical risk factors – one	123 (12.0)
Medical risk factors – two or more	21 (2.1)
Current smoker – yes	372 (36.4)

IQR *=* interquartile range, SD *=* standard deviation.

### 3.2. Travel characteristics and preventive practices

Participants were asked about travel history, preventive practices, and travel vaccination knowledge and attitudes (Table 2). In fact, 69.5% of our participants traveled within the last year, and the majority visited more than 1 country during the same year. Europe, Gulf region, and Africa were the most reported destinations.

**Table 2:** Descriptive summary of knowledge, attitudes, and practices.

Indicator	Value
Knowledge – aware of pretravel vaccination	786 (76.8%)
Knowledge – aware of preventive medications	576 (56.3%)
Knowledge – correctly rejects hypertension vaccine	987 (96.5%)
Attitude score (0–18) – mean (SD)	14.1 (3.3)
Attitude score (0–18) – median (IQR)	15 (12–17)
Practice – traveled in past year	711 (69.5%)
Practice – consulted a doctor before travel (of travelers)	74 (10.4%)
Practice – takes specific precautions	502 (49.1%)
Practice – carries a first aid kit	420 (41.1%)
Practice – takes medications while traveling	782 (76.4%)
Completed adult vaccination (strict definition)	395 of 881 (44.8%)

Higher attitude scores indicate a more pro-vaccine orientation. The hypertension item is a misinformation check (no such vaccine exists); the correct response is “No”.

IQR *=* interquartile range, SD *=* standard deviation.

Regarding preventive measures and precautions during travel, the data showed that 41.1% of participants carry first aid kits with them, while 76.4% bring emergency medications such as painkillers, antibiotics for travelers’ diarrhea, and antiseptics for wound care. In addition, 49.1% reported adopting other precautionary practices, including paying attention to hydration, food hygiene, or protection against insect bites. Table 3 details which precautions were prioritized by participants.

**Table 3:** List of precautions taken by travelers.

(what is the most important measure to be considered to avoid unfortunate consequences? is the most important to you)					
		Frequency	Percent	Valid percent	Cumulative percent
Valid		589	57.6	57.6	57.6
	Allergies prevention (needs to be explained)	7	.7	.7	58.3
	Food hygiene	105	10.3	10.3	68.5
	Food hygiene, insect bites prevention	3	.3	.3	68.8
	Food hygiene, medications for emergency or acute illness	1	.1	.1	68.9
	Food, water……..	51	5.0	5.0	73.9
	Food, water, insect bites……..	5	.5	.5	74.4
	Food, water, medications……	8	.8	.8	75.2
	Hand hygiene	6	.6	.6	75.8
	Infectious diseases prevention (explanation)	8	.8	.8	76.5
	Insect bites…..	88	8.6	8.6	85.1
	Medications and vaccines	42	4.1	4.1	89.2
	Travel insurance	2	.2	.2	89.4
	Water hygiene	108	10.6	10.6	100.0
	Total	1023	100.0	100.0	

### 3.3. Knowledge and attitudes toward travel vaccination

We then determined if travel vaccination was as important to them as other precautionary measures and if the respondents’ knowledge was adequate. Of the travelers in our group, 50.4% thought it was important to consult a physician prior to traveling; additionally, the time for consultation ranged from 1 to 4 weeks with 40% reporting 2 to 4 weeks as the right time for consultation and vaccination prior to departure (Table 4). However, only 10.4% sought the advice of their physician at any point before traveling.

**Table 4:** Time interval before consulting a travel clinic.

When should you consult your doctor?					
		Frequency	Percent	Valid percent	Cumulative percent
Valid	No answer	514	50.2	50.2	50.2
	1 wk	100	9.8	9.8	60.0
	2 wk	199	19.5	19.5	79.5
	4 wk	210	20.5	20.5	100.0
	Total	1023	100.0	100.0	

Moreover, 76.8% showed knowledge of pretravel vaccinations for certain parts of the world, and 56.3% were aware of preventive medications against certain infections while traveling.

27.3% of the participants reported concern about side effects from vaccines, 11% believed that vaccines themselves can trigger diseases such as diabetes or autism, and 8% believed that they contain harmful chemicals. The participants believed that the best way to spread awareness about travel medicine is mainly via social media (74.2%) as in Table 5, and only 0.8% thought it is important to consult a doctor.

**Table 5:** Strategies to promote travel vaccine awareness according to respondents.

What do you think is the best way of spreading awareness about adult and travel vaccination?					
		Frequency	Percent	Valid percent	Cumulative percent
Valid	Airlines, airports, travel agencies	16	1.6	1.6	1.6
	Awareness campaigns	10	1.0	1.0	2.5
	Billboards	66	6.5	6.5	9.0
	Brochures/flyers	53	5.2	5.2	14.2
	Doctor recommendations	8	.8	.8	15.0
	SMS	79	7.7	7.7	22.7
	Social media	759	74.2	74.2	96.9
	TV ads	5	.5	.5	97.4
	Videotapes in hospital	27	2.6	2.6	100.0
	Total	1023	100.0	100.0	

### 3.4. Psychometric properties of the attitude scale

The 9 attitude items, originally recorded as agree/ disagree/ uncertain, were recoded onto a 0–2 pro-vaccine metric (most pro-vaccine = 2, uncertain = 1), with negatively worded items reverse-scored so that higher totals consistently denote more positive attitudes. The resulting 9-item attitude scale demonstrated acceptable internal consistency (Cronbach α = 0.74). Exploratory factor analysis confirmed the data were suitable for factoring (Kaiser–Meyer–Olkin = 0.79; Bartlett test of sphericity χ^2^ (36) = 1694, *P* < .001) and yielded 2 interpretable dimensions: a vaccine-confidence factor (subscale α = 0.69) and a safety-concern/ hesitancy factor (subscale α = 0.64). The total score was left-skewed (skewness ≈ −0.94), reflecting a ceiling of generally positive attitudes. The 3 knowledge items were analyzed individually rather than as a composite: their internal consistency was poor (Cronbach α = 0.41), and the misinformation item was uncorrelated with the 2 genuine knowledge items, which is the expected and intended behavior. Because of the large sample, normality was assessed from skewness, kurtosis, and distribution plots rather than significance tests (which are oversensitive at n > 1000); nonparametric tests were therefore used as the primary bivariate approach.

### 3.5. Multivariable analyses

Each model included the a priori variables (age, gender, healthcare-worker status) together with factors reaching *P* < .20 in screening, entered simultaneously. For the practice outcomes, the attitude score and the 2 knowledge items were entered alongside the sociodemographic factors so that their independent (adjusted) effects could be estimated.

### 3.6. Determinants of attitude (linear regression)

The model was statistically significant but explained a modest share of variance (*R*^2^ = 0.085; *F*(16, 1006) = 5.81, *P* < .001). Healthcare-worker status was the strongest independent correlate of a more pro-vaccine attitude (*B* = 1.39, 95% CI: 0.92–1.87, *P* < .001). Current smoking was associated with a less positive attitude (*B* = −0.69, 95% CI: −1.13 to −0.26, *P* = .002), and graduate-level education with a lower score than school-level education (*B* = −1.16, 95% CI: −2.06 to −0.26, *P* = .011). Age, gender, having children, nationality, residency, and marital status were not independently associated with attitude. Collinearity diagnostics were satisfactory (all variance inflation factors < 5; Table 6).

**Table 6:** Multivariable linear regression – determinants of the attitude score.

Predictor	*B* (95% CI)	β	*P*
Healthcare worker	1.39 (0.92, 1.87)	0.19	<.001
Current smoker	−0.69 (−1.13, −0.26)	−0.10	.002
Education: graduate (vs school)	−1.16 (−2.06, −0.26)	−0.17	.011
Nationality: other (vs Lebanese)	0.94 (−0.11, 1.99)	0.05	.080
Age (per yr)	−0.01 (−0.04, 0.02)	−0.04	.401
Gender: female (vs male)	−0.10 (−0.52, 0.32)	−0.02	.633

*R*^2^ = 0.085. Model also adjusted for having children, residency, and marital status (all nonsignificant).

*B* = unstandardized coefficient, β = standardized coefficient, CI = confidence interval.

### 3.7. Travel practices (logistic regression)

A consistent pattern emerged across the 3 travel-practice models: the attitude score was not independently associated with any practice (all *P* > .57), whereas knowledge and gender were. Women had higher odds of taking precautions, carrying a first aid kit, and taking medications (all *P* ≤ .001). Knowledge of preventive medications was independently associated with all 3 practices, and knowledge of pretravel vaccination with taking medications (aOR 2.95, 95% CI: 2.01–4.34, *P* < .001; Table 7.

**Table 7:** Multivariable logistic regression – travel practices (adjusted odds ratios, 95% CI).

Predictor	Precautions	First aid kit	Medications
Female (vs male)	1.59 (1.21, 2.08)	2.15 (1.64, 2.83)	2.03 (1.47, 2.79)
Knowledge – preventive meds	1.65 (1.22, 2.22)	1.54 (1.13, 2.09)	1.61 (1.13, 2.31)
Knowledge – pretravel vax	1.63 (1.13, 2.34)	1.35 (0.93, 1.95)	2.95 (2.01, 4.34)
Attitude score (per point)	1.00 (0.97, 1.05)	1.00 (0.96, 1.04)	1.01 (0.97, 1.06)

Each model adjusted for age, gender, healthcare-worker status, and the sociodemographic factors retained on screening. Nagelkerke *R*^2^ = 0.09 (precautions), 0.12 (first aid), 0.15 (medications). Estimates whose 95% CI excludes 1.00 are statistically significant.

CI = confidence interval.

### 3.8. Pretravel consultation

Pretravel medical consultation could not be reliably predicted by the model. It explained only a very small part of the differences between travelers (Nagelkerke *R*^2^ = 0.03) and failed to correctly identify any of the 74 respondents who consulted. This suggests that seeking pretravel medical advice is influenced by factors other than travelers characteristics or their positive attitude toward vaccination.

### 3.9. Determinants of knowledge (logistic regression)

Both genuine knowledge outcomes were independently predicted by healthcare-worker status and higher education. Current smoking was associated with lower odds of pretravel vaccination knowledge (aOR 0.56, 95% CI: 0.40–0.78, *P* = .001; Table 8).

**Table 8:** Multivariable logistic regression – knowledge outcomes (adjusted odds ratios, 95% CI).

Predictor	Preventive medications	pretravel vaccination
Healthcare worker	2.92 (2.09, 4.09)	2.31 (1.49, 3.57)
Education: postgraduate (vs school)	2.58 (1.33, 5.03)	3.06 (1.52, 6.16)
Current smoker	0.87 (0.65, 1.17)	0.56 (0.40, 0.78)
Has children	0.67 (0.38, 1.17)	0.49 (0.24, 0.99)

Each model adjusted for age, gender, residency, marital status, and medical risk factors. Nagelkerke *R*^2^ = 0.16 (preventive medications), 0.17 (pretravel vaccination). Estimates whose 95% CI excludes 1.00 are statistically significant.

CI = confidence interval.

Overall, these findings indicate that practical knowledge, rather than favorable vaccine attitudes alone, was the strongest independent predictor of appropriate travel-related preventive behaviors.

## 4. Discussion

This study provides one of the first comprehensive assessments of travel vaccination knowledge, attitudes, and practices among Lebanese adults. Although participants generally demonstrated favorable attitudes toward travel vaccination and moderate awareness of travel-related preventive measures, uptake of pretravel medical consultation was low. Importantly, multivariable analyses showed that practical knowledge, rather than favorable attitudes alone, independently predicted appropriate travel-related preventive behaviors. These findings underscore the importance of knowledge translation and structured pretravel counseling in improving travel health practices.

Most participants in this study were Lebanese from different regions in the country, and educated. Their occupations varied from healthcare to business professions. This may enhance the representativeness of the sample, although caution remains warranted given the single-centered design.

The majority of the respondents had traveled within the past year, often to multiple destinations. Many repoted adopting basic preventive measures such as food and hygiene precautions carrying a first aid kit or bringing medication. These findings suggest general awareness of travel-related health risks. In addition, many participants acknowledged the importance of pretravel medical consultation.

Despite this, only 10.4% sought the medical advice before traveling. This gap suggests that awareness of travel vaccination and travel health risks does not necessarily translate into healthcare-seeking behavior. Several factors may contribute, including limited awareness of travel medicine services, underestimation of personal risk, logistical barriers, cost, and reliance on informal information sources. Vaccination guidelines are regularly updated and widely available in Europe and the United States.^[6,7]^ Although these resources can be easily accessed online; not everyone has access to this information, particularly those living in rural areas. Nevertheless, a study conducted in the United States in 2014 found that physicians saw an average of only 50 travelers per year for pretravel consultations.^[8]^ Similarly, a European study reported that while most travelers (73.3%) looked for tourist information about their destination, only half (52.1%) looked for travel health advice.^[9]^ Another study showed that among 1026,822 unique travelers departing from the United States only 6,12,795 (60%) travelers received travel vaccines.^[10]^

Another study reported that 51.9% of adults were not up to date on their vaccinations. The main reasons cited were lack of awareness (44.94%), the belief that vaccination was not necessary (48.8%), fear of side effects (3.6%), and financial concern (2.4%). Misconceptions such as the belief that vaccines contain harmful chemicals or can trigger diseases such as diabetes or autism may also discourage individuals from receiving recommended vaccines before traveling.^[11]^

These findings highlight the persistent influence of misinformation and the need for accessible, evidence-based communication delivered by trusted healthcare professionals and public health institutions.

Vaccine hesitancy has long been a major public health challenge and increased following the COVID-19 pandemic. Nevertheless, the pandemic also heightened awareness of infectious disease risks among travelers, underscoring the importance of travel medicine services, comprehensive pretravel counseling, and evidence-based vaccination recommendations.^[12]^ During the first half of 2021, 21% of adults in the United States and Australia reported vaccine hesitancy.^[13]^ The widespread dissemination of misinformation through social media has contributed substantially to this trend, highlighting the need for scientific and professional healthcare organizations to strengthen their presence on these platforms and promote accurate, evidence-based information.

Collaboration is essential to bridge these gaps in vaccine awareness. Governments, international organizations, healthcare providers, and the travel industry all have an important role in disseminating accurate information and promoting the value of travel vaccinations. Public health agencies can invest in awareness campaigns and educational programs to reach a broader audience, through social media being the preferred source of information among our population. These initiatives should also address the specific needs and concerns of different demographic groups. Ultimately, such efforts can help reduce the public health burden associated with endemic and travel-related infectious diseases.^[14]^

Furthermore, healthcare professionals are responsible for advising travelers about the vaccinations they may need based on their travel destinations, medical history, and individual risk factors. Current Centers for Disease Control and Prevention (CDC) travel medicine guidance recommends that every international traveler undergo an individualized pretravel risk assessment that includes destination-specific vaccination recommendations, malaria prevention when indicated, and counseling regarding food, water, insect, and environmental exposures. Recent evidence also suggests that awareness of travel-related infectious diseases remains variable even among urban populations, further highlighting the need for structured educational interventions before international travel. Accessible and reliable sources of information, such as national travel health websites and specialized travel clinics, can also provide valuable guidance and help travelers make informed decisions. A good example of such a resource is the travel health information provided by the Centers for Disease Control and Prevention through its disease directory.^[12,15,16]^

The present study provides several important insights into the relationship between attitudes, knowledge, and travel vaccination-related behaviors. Although participants generally demonstrated favorable attitudes toward vaccination, with the attitude scale showing a marked ceiling effect, positive attitudes alone were not sufficient to translate into preventive practices. In multivariable analyses, attitude scores were not independently associated with travel-related precautions, medication use, or first aid kit preparation, suggesting that knowledge and practical awareness may play a more direct role in shaping behaviors. Healthcare workers and individuals with higher educational attainment demonstrated greater knowledge of travel health recommendations, highlighting the importance of health literacy and access to reliable information. Interestingly, knowledge of preventive medications and pretravel vaccination was consistently associated with appropriate travel practices, emphasizing that targeted educational interventions may be more effective when they address specific knowledge gaps rather than focusing solely on vaccine acceptance. Recent travel medicine recommendations similarly emphasize that travelers benefit most when destination-specific vaccine recommendations are integrated with individualized risk assessment and pretravel counseling.^[17,18]^ The absence of strong predictors for pretravel consultation suggests that healthcare-seeking behavior before travel is influenced by additional factors, such as perceived need, accessibility of services, previous travel experience, or awareness of travel medicine services. These findings support the need for integrated travel health strategies that combine education, risk communication, and improved access to pretravel healthcare. This is consistent with international surveillance data demonstrating that many travelers continue to underutilize travel medicine services despite the availability of evidence-based preventive recommendations.^[19]^

Similar gaps between knowledge and practice have been described internationally. Studies among travelers in Europe, North America, and Australia have demonstrated that although travelers frequently seek destination-related information, fewer obtain professional travel health advice or recommended vaccinations before departure. A recent Australian study similarly demonstrated that many travelers possessed generally favorable views toward vaccination but still failed to seek pretravel vaccination or professional travel health advice, emphasizing that behavioral and logistical factors strongly influence vaccine uptake beyond positive attitudes alone.^[20]^ Recent evidence has also highlighted that vaccine confidence does not necessarily translate into vaccine uptake, as behavioral determinants such as accessibility, healthcare engagement, risk perception, and convenience strongly influence preventive practices. Our findings are consistent with this global pattern, demonstrating that positive attitudes toward vaccination alone were insufficient to predict preventive behaviors.

## 5. Limitation

This study has several limitations. First, it was conducted at a single academic medical center, which may limit generalizability to the entire Lebanese population. However, the hospital serves patients and visitors from different geographic and socioeconomic backgrounds. Second, the study period overlapped with the early COVID-19 pandemic, which may have influenced travel patterns and perceptions of infectious disease prevention. Third, the questionnaire relied on self-reported responses, introducing potential recall and social desirability biases. Finally, although the study assessed vaccine knowledge and attitudes, it did not explore in depth the individual motivations, barriers, financial factors, or healthcare access issues influencing travel vaccination decisions.

Future studies should explore specific barriers preventing travelers from seeking pretravel medical advice, including healthcare accessibility, cost considerations, perceived disease risk, and trust in healthcare recommendations. Qualitative studies may further clarify the reasons behind vaccine hesitancy and the factors influencing vaccination decisions among different population groups.

## 6. Future directions

Future research should evaluate interventions designed to improve travel vaccination uptake, including digital health interventions, social media-based education programs, primary care-based travel counseling, and community awareness campaigns. Longitudinal studies assessing whether improved knowledge translates into increased vaccination rates and safer travel behaviors would provide valuable evidence for clinical and public health decision-making.

## 7. Clinical implications

This study suggests that improving travel vaccination uptake requires more than increasing vaccine acceptance. The identification of knowledge gaps and the weak relationship between positive attitudes and preventive behaviors indicate that clinicians should incorporate structured travel health counseling into routine healthcare encounters, particularly for individuals planning international travel. Early identification of travelers requiring vaccination assessment may represent an important opportunity to improve preventive care.

From a public health perspective, these findings support the development of national travel health strategies in Lebanon. Such strategies may include standardized travel vaccination recommendations, improved public access to travel medicine services, collaboration with healthcare providers and travel agencies, and evidence-based communication campaigns through platforms frequently used by the population, particularly social media.

This study contributes new evidence regarding travel vaccination knowledge, attitudes, and practices in Lebanon, a population for which limited data are available. Unlike previous studies focusing mainly on vaccine acceptance, this study evaluates the relationship between knowledge, attitudes, and actual travel-related behaviors. Our findings demonstrate that favorable vaccine attitudes do not necessarily result in appropriate preventive practices, emphasizing the importance of addressing knowledge translation and healthcare accessibility.

## 8. Conclusion

This study demonstrates that Lebanese adults had generally favorable attitudes toward travel vaccination and moderate-to-good awareness of travel-related preventive measures. However, actual engagement with pretravel medical consultation and vaccination-related practices remained limited. Knowledge of preventive measures was more strongly associated with appropriate travel behaviors than vaccine attitudes alone. These findings highlight the importance of improving access to reliable travel health information and strengthening travel medicine services including integration of travel health risk assessment and vaccination counseling into primary care practice, particularly for individuals planning international travel.

## Author contributions

**Conceptualization:** Rania Sakr, Cima Hamieh, Mariana Helou, Alain Khouri, Rola Husni.

**Data curation:** Rania Sakr, Cima Hamieh, Rana Attieh, Alain Khouri, Remie Chrabieh, Rola Husni.

**Formal analysis:** Rania Sakr, Cima Hamieh, Mariana Helou, Remie Chrabieh, Ibrahim Ismail, Rola Husni.

**Investigation:** Rania Sakr, Cima Hamieh, Mariana Helou, Remie Chrabieh, Rola Husni.

**Methodology:** Rania Sakr, Cima Hamieh, Mariana Helou, Remie Chrabieh, Ibrahim Ismail, Rola Husni.

**Project administration:** Rania Sakr, Cima Hamieh, Mariana Helou, Remie Chrabieh, Rola Husni.

**Resources:** Rania Sakr, Cima Hamieh, Mariana Helou, Remie Chrabieh, Rola Husni.

**Software:** Rania Sakr, Cima Hamieh, Mariana Helou, Remie Chrabieh, Rola Husni.

**Supervision:** Mariana Helou, Rola Husni.

**Validation:** Rania Sakr, Cima Hamieh, Mariana Helou, Remie Chrabieh, Rola Husni.

**Visualization:** Rania Sakr, Cima Hamieh, Mariana Helou, Remie Chrabieh, Rola Husni.

**Writing – original draft:** Rania Sakr, Cima Hamieh, Rana Attieh, Alain Khouri, Remie Chrabieh.

**Writing – review & editing:** Rania Sakr, Cima Hamieh, Mariana Helou, Rola Husni.




