# Supplementary material for: How Lebanese adults perceive travel vaccinations: A single center study
Source: Medicine (Baltimore). 2026 Jul 31;105(31):e50062. doi: 10.1097/MD.0000000000050062 (PMC13433072; doi:10.1097/MD.0000000000050062)
Supplement: Supplementary file 2 [file medi-105-e50062-s002.docx]

ASSESSMENT OF GENERAL POPULATION KNOWLEDGE ABOUT TRAVEL MEDICINE AND ROUTINE ADULT VACCINATION

l- Demographic data

1-Date of Birth: / /

2-Gender: □Male □Female: Pregnant □No □Yes □Possible

3-Level of education: □No formal education □High school □Bachelor, which?_____________
 □Master, which?_____________ □Doctoral, which?_____________

4-Area of residency: □South □North □Bekaa □Beirut □Mount Lebanon
 □Nabatieh □Baalbek-Hermel □Akkar □Abroad: _____________

5-Occupation: □Healthcare: ___________ □Other: __________ □Unemployed

6-Marital status: □Single □Married □Divorced □Widowed

7- Number of children if present:_______________

8- Nationality:________________

II- About Adult Vaccination

Please reply to the following statements about vaccination in adults (18 years and older)

1-Do you have any chronic medical condition?

□Chronic lung disease □Asthma □Diabetes □Chronic kidney disease □Coronary artery disease
□Heart failure □Cancer □Pregnancy □On Steroid treatment □Chronic liver disease □Alcoholism
□Other__________________

2-Do you smoke?
□ Yes □ No

3-Have you ever been recommended vaccination in your adult life?
□ Yes, by whom?______________________________________________________
 and which vaccines?_____________________________________________
□ No

4-Have you completed your vaccinations as an adult?
□ No, why?_______________________________________________________
□ Yes, which?_______________________________________________________

5- Does your job require you to be vaccinated?
□ Yes □ No

6-All adults, regardless of their health status, must update their vaccination (booster or new vaccines)

□ Agree □ Disagree □Uncertain

7-Vaccines are highly protective against the diseases they are targeting
□ Agree □ Disagree □Uncertain

8-Vaccines may trigger some diseases such as diabetes, autism or other, as side effects
□ Agree □ Disagree □Uncertain

9-Vaccines are unnecessary because we can treat the disease once it occurs
□ Agree □ Disagree □Uncertain

10-Vaccines contain chemicals that are harmful to humans
□ Agree □ Disagree □Uncertain

11-Vaccines are mainly being promoted because they are of economic benefit to pharmaceutical companies
□ Agree □ Disagree □Uncertain

12-I am concerned about the side effects of vaccines
□ Agree □ Disagree □Uncertain

13-If there were any indicated vaccines, I would get them done
□ Agree □ Disagree □Uncertain

14-If there were any indicated vaccines, I would recommend to others
□ Agree □ Disagree □Uncertain

15-Vaccines are of less importance in adulthood
□ Agree □ Disagree □Uncertain

III- About Travel Medicine

1-Have you traveled the last year?
□ Yes, where to___________________________________ □ No

2- Have you consulted your doctor before traveling?
□ Yes □ No

3-Do you think you should consult a doctor before traveling?
□ Yes, when? □1 week before □2 weeks before □ 4 weeks or more before

□ No

4-Do you take a first aid kit while travelling?
□ Yes □ No

5-Do you take medications with you while traveling?
□ Yes □ No

6-Do you take specific precautions while travelling (water, food, insect bite..)?
□ Yes , which one is the most important for you? ________________________
□ No

7- Are you aware about preventive medications against certain infections while travelling?
□ Yes □ No

8- Do you know about pre travel vaccination for certain parts of the world?
□ Yes □ No
